# Editorial: Epithelial to Mesenchymal Plasticity in Colorectal Cancer

**DOI:** 10.3389/fcell.2022.950980

**Published:** 2022-06-23

**Authors:** Federico Bocci, Regine Schneider-Stock, Sreeparna Banerjee

**Affiliations:** ^1^ NSF-Simons Center for Multiscale Cell Fate Research, Irvine, CA, United States; ^2^ Department of Mathematics, University of California, Irvine, Irvine, CA, United States; ^3^ Experimental Tumorpathology, Institute of Pathology, Universitätsklinikum, Erlangen, Germany; ^4^ Comprehensive Cancer Center-EMN (CCC), Friedrich-Alexander University Erlangen, Erlangen, Germany; ^5^ Department of Biological Sciences, Middle East Technical University, Ankara, Turkey

**Keywords:** EMT, colorectal cancer, plasticity, partial EMT, MET

Epithelial-mesenchymal transition (EMT) is a core cellular program in vertebrate embryonic development where the expression of key junctional and cytoskeletal proteins such as E-Cadherin and Vimentin orchestrate stages of embryogenesis such as gastrulation or morphogenesis (Kalluri and Weinberg, 2009; Hanahan and Weinberg, 2011). In 2003, EMT was linked to carcinogenesis (Yang et al., 2020) leading to extraordinary interest in understanding the molecular basis of EMT in cancer.

When epithelial tumors progress, they lose cell–cell contacts and apico–basal polarity, transforming into spindle-shaped mesenchymal structures. Such cells also gain capabilities of motility and invasiveness for metastasis (Dongre and Weinberg, 2019). At metastatic sites these cells can revert to an epithelial state to proliferate and generate secondary metastases, a process defined as mesenchymal–epithelial transition (MET). Due to this ability to undergo both EMT and MET, tumor cells can also exist in a number of intermediate states, the so called epithelial/mesenchymal hybrid phenotypes contributing to epithelial to mesenchymal plasticity (EMP) (Saitoh, 2018). Such cells maintain cell-cell contact to disseminate as circulating tumor cells (CTC) (Jolly et al., 2015). Although single cell CTCs appear to be more predominant, clustered CTCs (also known as microemboli) are associated with more efficient metastatic spread and worse prognosis in most carcinomas (Mizukoshi et al., 2020; Semaan et al., 2021). E-cadherin is gained in these cells; however, such collective migration is thought to reflect intermediate EMT.

EMT proteins can have various non-EMT related functions and act in a tissue-specific manner such as acquisition of immunosuppression and cancer stem cell (CSC)-like features (Mani et al., 2008; Saitoh, 2018). The tumor-associated reactive stroma is also known to actively regulate the expression of EMT genes (Dongre and Weinberg, 2019) and thereby promote tumor progression and metastasis. In this special issue, Vuletic et al. review the role of Natural Killer (NK) cells in the process of EMT (Vuletic et al.). EMT-associated gain of NK cell ligands in cancer cells may enhance NK cell mediated cytotoxicity, whereas an immune suppressive tumor microenvironment (TME) may activate enzymes that lead to a loss of ligands. He et al. show that the expression of PSMC5 altered the type of immune cells in the TME of CRC by suppressing the infiltration of CD8^+^ T cells and enhancing the infiltration of innate immune cells such as macrophages and neutrophils and was associated with EMT (He et al.).

EMT may enhance chemoresistance with evidence for enhanced drug efflux, slower cell proliferation and avoiding apoptosis signaling pathways. Slug was implicated in the development of partial EMT and resistance to doxorubicin via upregulation of drug efflux transporters and stemness in liver cancer cell lines (Karaosmanoğlu et al., 2018). Yu et al. show in this special issue that Vitamin D3 can enhance the sensitization of CRC cells to ionizing radiation via the expression of cystatin D and plasminogen activator inhibitor-1 (PAI-1) and by reversing EMT (Yu et al.). Song et al. show that expression of β-arrestin1 enhanced the motility of CRC cells via the activation of Wingless/integration-1 (Wnt)/β-catenin signaling (Song et al.).

Reliance on one or two genes to evaluate a process as complex as EMT as well as the use of acute tumor models may not be sufficient to recapitulate the heterogeneity, metabolic idiosyncrasies, and growth pattern of a slow growing tumor (Wang et al., 2016). Over the last decade, integrating experimental investigations with theoretical and computational modeling have helped uncover the dynamics of EMT and characterize intermediate epithelial/mesenchymal cell states. These approaches have been able to provide significant clinical insights by predicting critical molecular players and interventions and their effect on the stability of highly aggressive intermediate E/M phenotypes (Steinway et al., 2015; Bocci et al., 2019b). The rapid expansion of single cell transcriptomics has also brought forth integrated approaches to identify intermediate states along EMT trajectories (Sha et al., 2020; Sacchetti et al., 2021), reconstruct core gene regulatory networks (Ramirez et al., 2020), and study intra- and intercellular signaling dynamics during EMT (Bocci et al., 2021). While these methods have provided invaluable insights, many challenges lie ahead that will benefit from the integration of experimental investigation with theoretical and computational analysis.

A first area of investigation relates to the connection between EMT and other axes of cancer progression, such as initiation, resistance to therapies, tumor-immune system interactions and metabolism. Mathematical models of the underlying interconnected regulatory networks suggest that hybrid epithelial/mesenchymal cancer cells exhibit a high phenotypic plasticity that enables stem-like properties (Bocci et al., 2019a). Using modeling experiments, Jia et al. report in this special issue that NRF2 mediated epigenetic regulation of the expression of Snail can stabilize a cell in the state of partial EMT, preventing it from transitioning to a complete mesenchymal state (Jia et al.). Moreover, modeling experiments suggest that partial EMT can facilitate hybrid metabolic states with combination of glycolysis and oxidative phosphorylation, contrary to primary tumors that mostly rely on glycolysis (Jia D. et al., 2021). These findings need to be supported by robust *in vivo* data in order to identify metabolic vulnerabilities that can be targeted for therapy.

A second open area of investigation concerns the integration of biochemical and biophysical aspects of EMT. While the models discussed above adopt a “systems-biology” approach that focuses primarily on the intracellular circuitry at the bases on EMT regulation, cells undergoing EMT modify their mechanical properties by regulating their adhesion, polarity, and cytoskeletal structure (Dongre and Weinberg, 2019), suggesting the possibility of high heterogeneity. Adding to this complexity, the regulation of EMT depends on the interplay between tumor cells and their local microenvironment, for instance by regulating the ability of leader cancer cells to ease the passage of follower cells through the extracellular matrix (Mercedes et al., 2021). Therefore, cancer cell migration and invasion rely on better understanding of the intricate mechanochemical feedback between cancer cells and their microenvironment.

This special issue on “Epithelial to Mesenchymal Plasticity in Colorectal Cancer” explores the complex ramifications of these themes with a specific focus on colorectal cancer (CRC). The results reported provide new insights into EMT plasticity and its implications in CRC.
